# Continuously Tunable, Polarization Controlled, Colour Palette Produced from Nanoscale Plasmonic Pixels

**DOI:** 10.1038/srep28062

**Published:** 2016-06-17

**Authors:** Eugeniu Balaur, Catherine Sadatnajafi, Shan Shan Kou, Jiao Lin, Brian Abbey

**Affiliations:** 1 Australian Research Council Centre of Excellence for Advanced Molecular Imaging, Australia; 2 Department of Chemistry and Physics, La Trobe Institute for Molecular Science (LIMS), La Trobe University, Victoria 3086, Australia; 3 School of Engineering, RMIT University, Melbourne, Victoria 3001, Australia

## Abstract

Colour filters based on nano-apertures in thin metallic films have been widely studied due to their extraordinary optical transmission and small size. These properties make them prime candidates for use in high-resolution colour displays and high accuracy bio-sensors. The inclusion of polarization sensitive plasmonic features in such devices allow additional control over the electromagnetic field distribution, critical for investigations of polarization induced phenomena. Here we demonstrate that cross-shaped nano-apertures can be used for polarization controlled color tuning in the visible range and apply fundamental theoretical models to interpret key features of the transmitted spectrum. Full color transmission was achieved by fine-tuning the periodicity of the apertures, whilst keeping the geometry of individual apertures constant. We demonstrate this effect for both transverse electric and magnetic fields. Furthermore we have been able to demonstrate the same polarization sensitivity even for nano-size, sub-wavelength sets of arrays, which is paramount for ultra-high resolution compact colour displays.

Extraordinary optical transmission (EOT) from subwavelength apertures fabricated in thin metallic films was first observed by Ebbesen and co-workers^1^,^2^ in the late 1990’s. Since then the number of devices which have been developed to exploit this effect, in order to control zero-order light at the nanoscale has grown substantially. This interest is partly motivated by the fact that subwavelength apertures have been found experimentally to transmit more light than is predicted by classical diffraction theory^3^. The EOT effect is correlated to the resonant excitation of surface plasmons (SP) due to the coupling of incident radiation at the metal-dielectric interface and localized SPs related to the aperture geometry^4^. This has led to a large number of studies being published that explore the effect of geometry and material characteristics on the optical properties of the transmitted light. The results from this work have facilitated the use of subwavelength apertures in a range of applications, from nanoscale bio-sensors^5^,^6^,^7^,^8^ to colour filters^9^,^10^,^11^,^12^,^13^,^14^,^15^,^16^ and metadevices^17^.

Of particular interest is the high sensitivity of these devices which allows the detection of small changes in the dielectric constant within the near-surface region. This opens up a number of promising applications in the field of bio-sensing, where *in-vivo* diagnostics is essential for non-invasive detection^5^,^7^,^18^. For example, Yanik *et al.* demonstrated high-efficiency biosensing of live RNA viruses using arrays of circular apertures which correlate resonant peak shifts to near-surface dielectric changes arising from the selective attachment of the virus to particular anchor sites on the metal surface^8^. Sub 1 ng/mL levels of IgG protein have also been detected using the same approach demonstrating fast, label-free detection without the need for fluorescent markers^19^,^20^. In addition, Lee *et al.* were able to image gliding mycoplasma mobile via EOT through arrayed nanoholes^21^.

Advances in nano-fabrication techniques have enabled the realization of ever more complex apertures, allowing for the intricate manipulation of light^22^,^23^,^24^,^25^,^26^. When compared to more elaborate aperture designs, there are several key limitations that exist for circular aperture-based plasmonic devices. 1. They are fundamentally insensitive to the polarisation direction for linearly polarized light^27^; this prevents their use in the polarization-induced manipulation of light or for the direct imaging of polarization induced states. 2. Using circular apertures, the resonant frequency is sensitive to both the periodicity of the holes as well as their size^15^,^16^,^28^. This limits the smallest achievable device size and implies the need for specific methods to be developed to correct for ‘stitching’ artefacts when arrays of circular apertures are used to create pixels in colour displays^29^. These methods are not straightforward to implement and require considerable effort to realize effective colour separation. 3. The EOT efficiency is poorer for circular apertures compared to rectangular apertures, imposing a limit on the source intensity and sensitivity^30^. These limitations have driven the research and development of optical plasmonic devices with different geometries e.g. coaxial^31^,^32^, elliptical^33^, square, rectangular and cross-shaped^10^,^30^,^34^,^35^. In the present study we focus on cross-shaped aperture arrays, exploiting their inherent polarisation sensitivity to demonstrate continuous control of the transmitted spectrum over the entire visible range. Furthermore, we demonstrate that full functionality is retained from individual plasmonic ‘pixels’ of just a few hundreds of nanometres in size.

Polarization-responsive plasmonic devices have sparked particular interest as fundamental components in wave plates^36^, focusing devices^37^ and dynamic colour filters^12^,^38^. Elliptical, rectangular and cross-shaped nano apertures are the primary candidates for such devices due to their inherent broken symmetry and their ability to support localized surface plasmon (LSP) states. Cross-shaped apertures are currently the leading contender as the basis for polarisation-responsive plasmonics since they can uniquely respond to multiple polarization states with comparable efficiency. The polarisation properties of cross-shaped structures fabricated on glass substrates have previously been studied by Ellenbogen *et al.*^38^. Their study showed that modifications to the transmitted colour palette arise due to the interaction of incident light and the LSP states induced inside the cross-shaped structures under certain polarisations. It is important to note that this process of colour filtering is conceptually different in circular aperture-based systems, where EOT plays a predominant role in the light propagation through the apertures. A number of publications have been devoted to understanding and developing theories to describe the processes responsible for the higher efficiencies and polarization-induced response of such structures^34^,^35^,^39^,^40^,^41^. The majority of these studies rely solely on numerical modelling, but in general they conclude that surface plasmon polaritons (SPPs), LSPs, Wood-Rayleigh anomalies (RA) and Fabry-Perot interference of cavity modes can be used to describe the light interaction with these structures. The cross-shaped aperture arm length and width, in particular, has been found to play a critical role in determining the device optical transmission properties^34^,^35^. In a very recent study by Li *et al.* such cross-shaped apertures were shown, for the first time, to be capable of producing two-colour transmission^42^ (published at the time the present work was first submitted). Here we show that using cross-shaped apertures in combination with polarization control it is possible to generate not only two-colour spectra but a *continuously* tunable colour palette (ranging from violet through to red) which is a key step for applications including high-resolution chemical sensing and miniaturised displays. We also present a theoretical and simulation study of these devices and are able to understand and replicate the key fundamental features of the resulting spectra.

Using high-accuracy nanofabrication protocols in combination with finite element modelling (FEM) calculations, we explore here the application of cross-shaped apertures as polarisation sensitive colour filters for light in the visible range of the spectrum. A key finding in the present study is that we are able to produce colour changes, simply by varying the pattern periodicity in the X and Y directions, whilst keeping the geometrical size of the crosses constant (Fig. 1). It is also important to contrast the results here with the extensive colour filtering work carried out using circular apertures. In the circular aperture array case the transmitted spectrum cannot be varied using the incident polarisation and the colour filtering properties are dependent on both the periodicity and size of the holes. For example, smaller holes will result in a shift of the visible spectrum towards blue, whilst larger holes shift the transmitted spectrum towards red. We have also explored, for the first time, the smallest size which it is possible to make individual plasmonic, full-colour, nanopixels whilst maintaining complete functionality. Finally, using analytical expressions for LSPs, SPPs and RAs, we present a detailed theoretical study exploring the origin of the resonant peaks and how they relate to the shape and periodicity of the apertures. The experimental results are compared directly to FEM numerical calculations which confirm the dependence of the resonant peak position on the periodicity. In summary, the key findings of the present study are as follows:

Exploring combinations of periodicity and incident polarisation we generate a continuously tunable full colour spectrum; previously using polarisation control only two distinct colour spectra have been demonstrated^40^. Full spectral control is critical for applications such as miniaturised colour displays^9^,^13^,^16^,^28^.

We have determined the minimum size possible for plasmonic nanoscale pixels using cross-shaped apertures. Surprisingly we find full functionality is maintained all the way down to just 2 × 2 crosses giving a smallest pixel size of just 280 nm. This finding could have important applications in, for example, the localised detection of single molecules previously demonstrated using isolated nanoparticles of Au or Ag^43^,^44^.

In grating based systems colour filtering is determined via SP modes in the TM orientation and by a waveguide mode in the TE^10^ orientation and in circular apertures it is determined by both aperture size and periodicity. Here however, continuous colour variation can be achieved with a fixed aperture geometry simply by varying the pattern periodicity. We have also explored the effect of varying the cross arm lengths, however since this is not required for full spectral control and is described in ref. 40, the results are not included here.

We have developed an analytical model supported by FEM analysis that explains and predicts the origin of resonant peaks and their dependence on the array periodicity in the visible range. This was previously well-understood for circular apertures but, until now, has not been examined in detail for cross-shaped apertures^1^,^2^,^45^.

## Results

### Effect of array periodicity and polarisation on transmission

**Figure 1:**
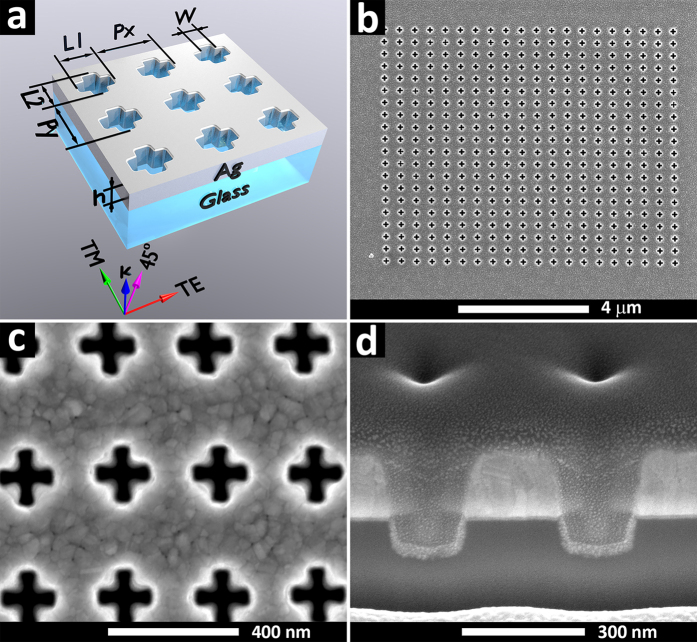
(**a**) Design of cross-shaped aperture arrays. Linearly polarized light is incident from the glass side and the transmitted spectra are collected from the metal film side. (**b**) An SEM image of a typical aperture array. (**c**) A detailed image of the array. (**d**) FIB cross-section across the length of the arms.

**Figure 2:**
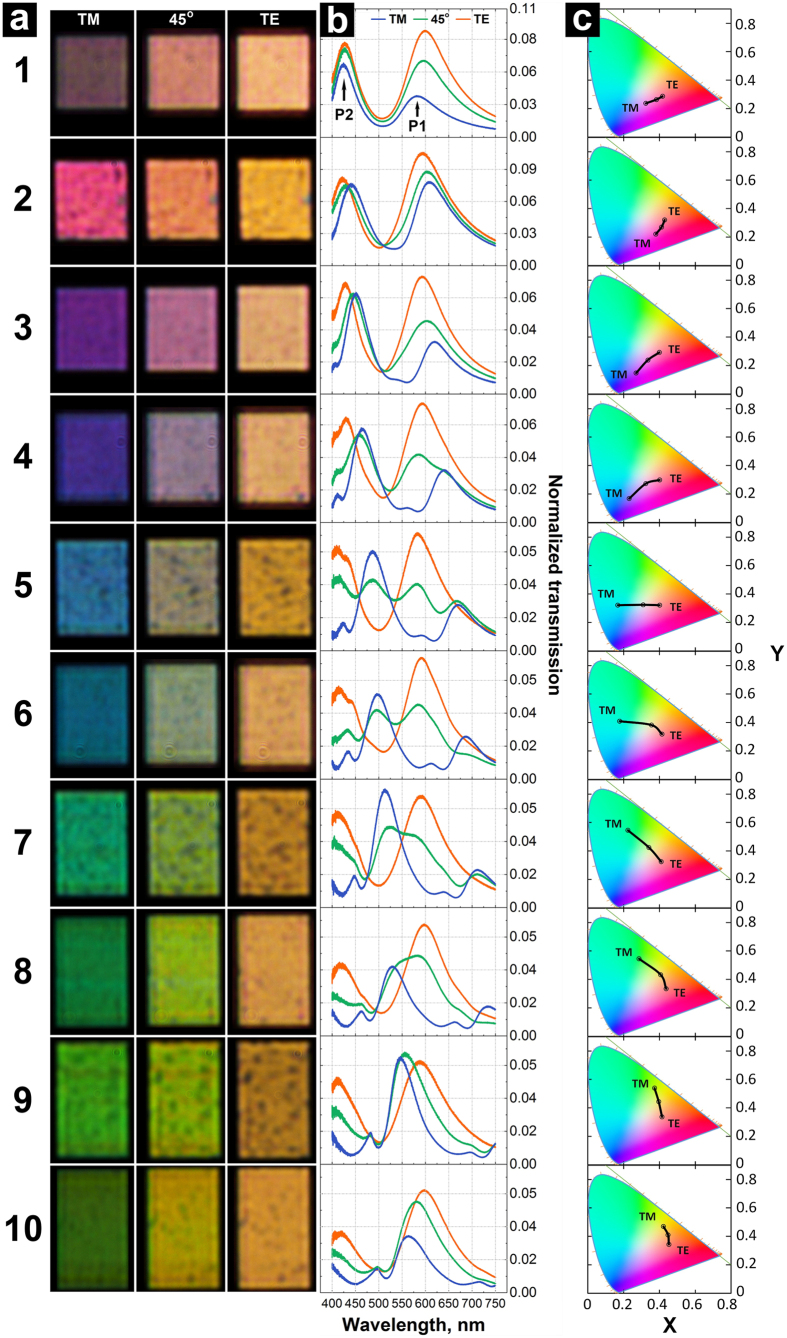
(**a**) Optical images of transmitted light for ten different periodicities using TM, 45° and TE polarized light. (**b**) Associated transmission spectra normalized to the incident light showing the position of the main peaks P1 and P2. (**c**) Experimental transmission spectra mapping of (**b**) to points on the CIE 1931 xy chromaticity diagram, demonstrating active polarization-dependent color tuning.

Figure 1a shows the design of the cross-shaped aperture arrays used in this study denoting the incident wave vector *k* from the glass side and the TM, 45° and TE polarisation vectors with respect to the aperture array. Figure 1b shows an SEM image of a representative array of cross-shaped apertures after milling with Fig. 1c demonstrating the high quality of the patterns. Figure 1d shows an SEM image of a FIB cross-section through the length of the arms showing the quartz substrate, Ge and Ag layers. In order to achieve a clean cut, Pt/C e-beam induced deposition was used to cover the device. The cross-section of these structures reveals the intrinsic footprint of ion milling–the characteristic bevelled profile of the patterns. This effect cannot be entirely eliminated and can only be minimised by using low currents during the milling process. The figure also shows that the substrate was also partially milled. Ten arrays of patterns were fabricated using different periodicities in the Y direction ranging from 300 to 480 nm in 20 nm steps while keeping the same periodicity of 280 nm in X direction. The arm length and width were kept at ~160 and 40 nm respectively. Figure 2a shows the optical images that were collected for all samples illuminated with TE, 45° and TM linearly polarized light.

As the periodicity increased, the whole palette of colours could be detected using TM polarized light. However, as the polarisation was changed to the TE mode, a single colour dominated the spectrum as the periodicity in X direction was kept constant. Optical images using 45° polarized light show a mix of TM and TE polarized colours. We found that any polarisation between TM and TE polarized light led to a gradual colour change from one polarisation to another. The footprint of such effects can be clearly seen in the 45° polarized light spectra, where a combination of TM and TE peaks are visible. Transmission spectra (Fig. 2b) illustrate the peaks associated with TM, 45° and TE polarisation. The nature of the individual peaks will be described later in detail. Overall, the transmission spectra of the TM polarized light reveals a trend of the peak shift towards the red region of the spectra as the periodicity in the Y direction is increased, while it remains almost unchanged using TE polarized light. Transmission spectra at 45° polarisation reveals the convolution of the TE and TM modes. By gradually changing the distance between the apertures in one direction whilst keeping it constant in another direction, a rainbow-like colour palette was achieved. In order to explicitly highlight the influence of polarisation control on the transmitted light, linear colorimetric transformations were applied to each spectrum. This allowed each polarization-angle dependent transmission spectrum to be mapped to a single point in the CIE 1931 xy chromaticity diagram. The corresponding results for each sample (with varying periodicity) are shown in Fig. 2c. The palette of colors produced by a particular sample is defined by a line on the CIE 1931 xy chromaticity diagram and is a result of the interpolation of the TE and TM modes at a particular polarization angle. The TE and TM points on the plot can be adjusted by changing the periodicity of the cross-shaped aperture array.

**Figure 3:**
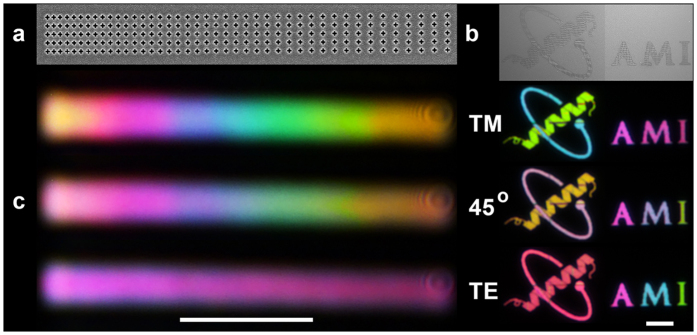
(**a**) SEM image of an array of apertures with varying the periodicity in the X-direction. (**b**) SEM image of a logo fabricated by using apertures placed at different periodicities. (**c**) Corresponding optical images of transmitted light using TM, 45° and TE polarized light. The scale bars are 5 and 15 μm respectively.

Figure 3a,c illustrate this effect with 3a showing the SEM image of an array consisting of 5 × 42 cross-shaped apertures and 3c the corresponding optical images under TM, 45° and TE polarisations. Figures 3b,c demonstrate that using this principle, more complex structures can be readily accomplished. From an application standpoint it is important to note the fact that even subwavelength nanometer-size features are able to consistently reproduce a similar colour palette and polarisation sensitivity.

**Figure 4:**
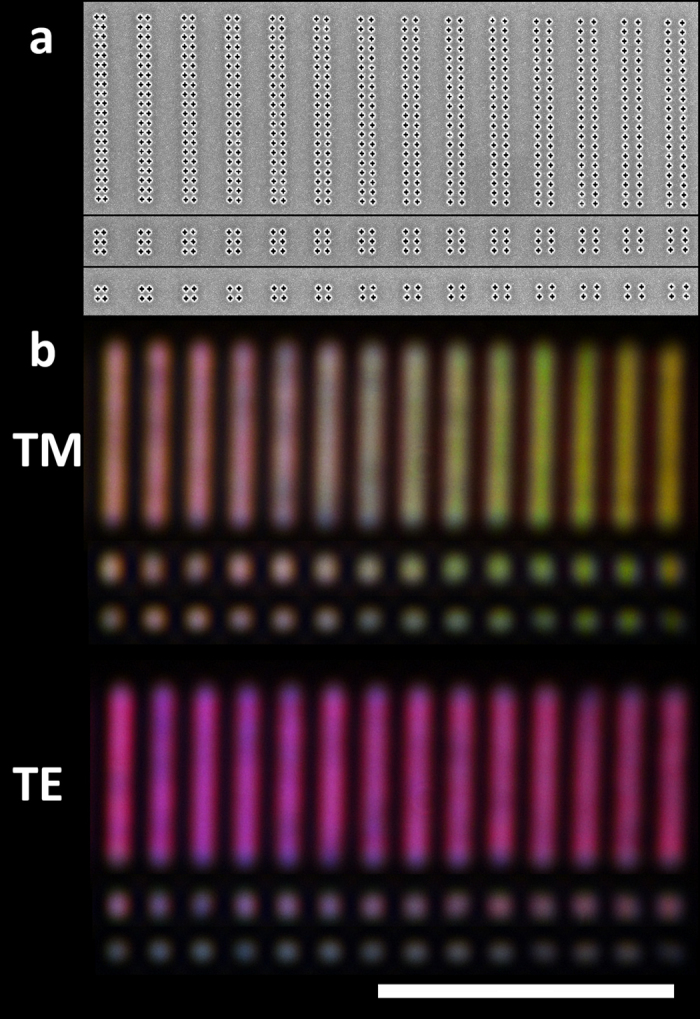
(**a**) SEM images of an array of apertures (2 × 20, 2 × 3 and 2 × 2) with varying the periodicity in the X - direction. (**b**) Corresponding optical images of the transmitted light using TM and TE polarized light. The scale bar is 10 μm.

Figure 4 shows SEM images of 2 × 20, 2 × 3 and 2 × 2 arrays (Fig. 4a) and their resultant optical images under TM and TE polarisation (Fig. 4b). The pixel size of the 2 × 2 aperture arrays is just 280 × 280 nm and 280 × 500 nm for the smallest and largest periodicity respectively.

**Figure 5:**
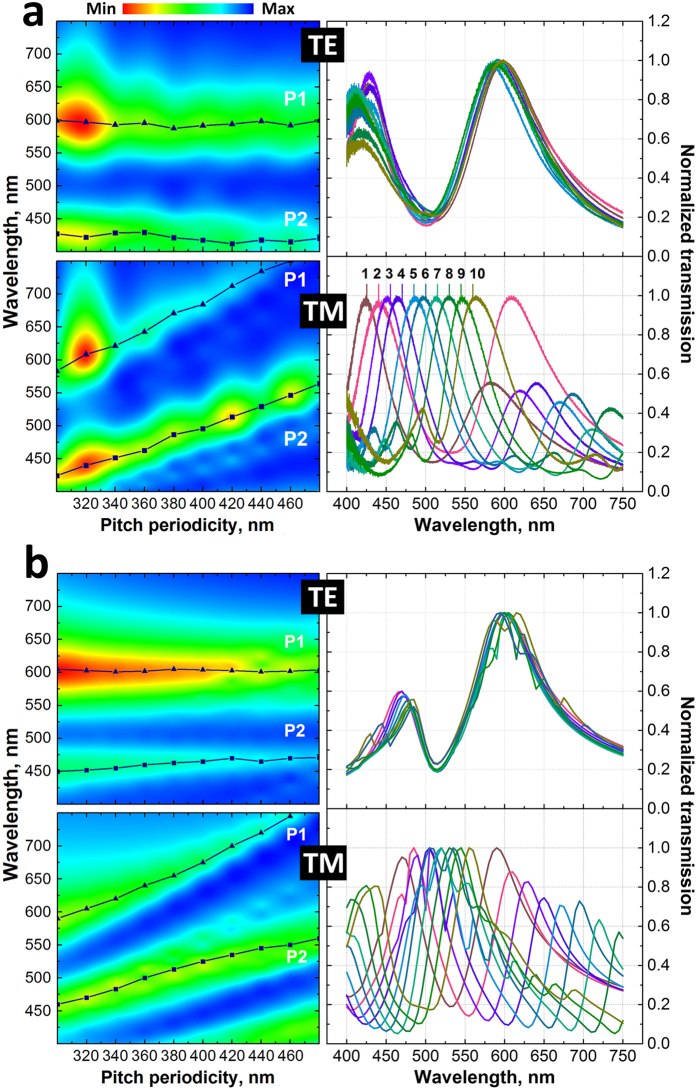
Intensity maps of the transmitted light as a function of periodicity for TM and TE polarized light and their associated transmission spectra normalized to the maximum value for experimental (**a**) and calculated (**b**) data. P1 and P2 plots indicate the position of main peaks.

Figure 5a,b shows the transmitted intensity maps and a summary of the transmission plots of the experimental and simulated data accordingly for TE and TM polarisations as a function of periodicity. The intensity maps also include the maximum position of the main peaks (*P1* and *P2*). Both figures conclusively show the peak position wavelength increases as the periodicity of the apertures increases covering almost the full range of the visible spectrum.

### Numerical simulation and analytical interpretation of plasmonic modes

Experimental data was validated against FEM simulations performed in COMSOL Multiphysics 5.1 using the Radio Frequency module^46^. When designing the model geometry, we have taken into account irregularities of the aperture induced by the FIB fabrication process such as: bevelling, edge and corner roundness and over-milling. These effects can clearly be seen in Fig. 1c,d. Such refinement of the model tends to produce less artefacts in the simulated data and provide better agreement with the experimental data. Figure 5b shows the calculated transmitted intensity maps and provides a summary of the transmission plots for TE and TM polarisations as a function of periodicity illustrating good agreement with the experimental data.

Transmission of the incident light through the apertures is an effect associated with the various modes that are induced by the incident electromagnetic field interacting with the metal film resting on a dielectric. These modes can be correlated to a whole range of phenomena including: SPPs at the dielectric/metal interface, LSPs (or cavity modes) inside the aperture and RAs associated with the metal grating formed by the aperture resting on a dielectric. Fabry-Perot resonances due to the shape and thickness of the apertures^2^,^34^,^41^,^47^ have also be observed, however they are only expected to occur when the film thickness *t* >λ/2, which is not the case here (where *t *= 150 nm). Therefore, the origin of the peaks can be mainly ascribed to three different modes that interfere (constructively or destructively): SPPs, LSPs and RAs, resulting in a set of maxima and minima in the transmission spectra.

The generalized dispersion equation of resonant peaks for different Bloch modes of SPPs at normal incidence, which is widely used for periodic apertures in metallic films is^2^:


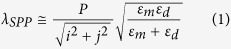


where *P* is the array periodicity, *i* and *j* are integers corresponding to the scattering orders from the aperture array, *ε*_*m*_ and *ε*_*d*_ are the permittivity of the metal and dielectric medium respectively. When calculating these resonances for different modes, we took into consideration the dependency of metal permittivity on the wavelength^48^. As a linearly polarized light source was used, the array periodicity values were taken in the direction perpendicular to the polarized incident vector. Equation 1 however, doesn’t account for the presence of apertures and the associated scattering losses, therefore, the experimental values of resonant wavelengths tend to be red-shifted. This red-shift has been experimentally observed and analytically calculated using the Kramers–Kronig relation^49^ which occurs at ~4% red-shifted wavelengths.

Furthermore, for metal films with thicknesses greater than two times the skin depth of SPP propagation in metal (which is ~29 nm for silver at 400 nm incident wavelength)^50^, no coupling between SPP modes on the glass/silver and air/silver interface can occur^45^. This means that two distinct sets of features associated with these modes are expected in the transmission spectra.

Moreover, scattering modes that satisfy the RA condition is described as:


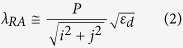


**Figure 6:**
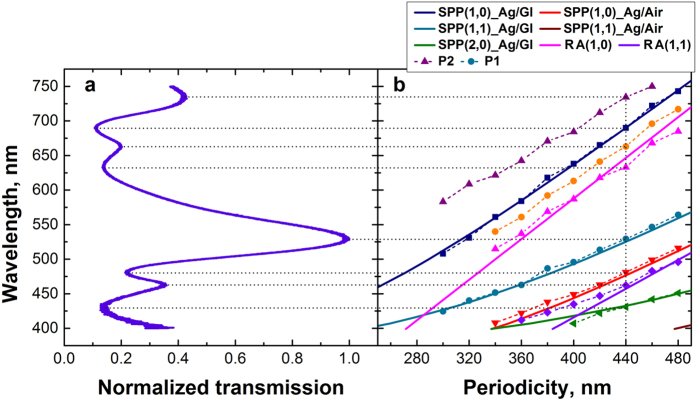
(**a**) Normalized transmission spectra for the 440 nm periodicity array (sample S8) showing the correlation of the peaks’ origin of different modes. (**b**) Experimental (doted lines) and calculated (full lines) SPP and RA modes.

All SPP modes in the measured spectral range associated with Ag/glass and Ag/air interfaces along with RA modes were calculated for different array periodicities and compared to the experimental data (Fig. 6b).

An almost a perfect agreement between the experimental data and the calculated values is observed, which gives a clear indication of the origin of the associated peaks. For each increase in the periodicity, the SPP and RA modes are expected to shift by:


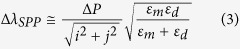



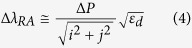


For example for a 20 nm increase, the Δ*λ* of (1, 0) SPP Ag/glass and (1, 0) RA modes increases by ~25 nm and 31 nm respectively. It is interesting to note that all (1, 0) and (2, 0) SPP modes and (1, 0) RA modes produced minima in the spectra, whilst (1, 1) SPP and (1, 1) RA modes produced maxima. Earlier studies of the transmission through such structures did not reveal the presence of these satellite peaks^39^,^41^. This might be attributed to a high degree of imperfection during the fabrication process in previous studies or to the limited data available for a wide range of periodicities leading to a featureless broad peak at resonant frequencies. Therefore, the correlation of the peaks have been largely associated with the fundamental modes of SPPs and RAs. Both our experimental results and theoretical calculations show splitting of the peaks associated with the (1, 0) SPPs at the Ag/air interface and (1, 1) RA and (2, 0) SPP modes at the Ag/glass interface for an array periodicity of between 320–340 nm (Fig. 6b). The second local maxima situated at higher wavelengths also occurs for the same periodicity. Such satellite peaks have previously been observed only for circular arrays^2^ and were attributed to fundamental modes of SPPs and RAs for different dielectric/metal film interfaces. In that case, the SPPs were correlated to the maxima in the transmission spectra, while RAs were associated with the minima. Conversely, recent studies on cross-shaped structures in the mid to far infrared regions, correlated the minima to the destructive influence of the SPPs and LSPs associated with the structures, and the maxima to LSP resonances^41^. In our study, the origin of the second peak (*P2*) can be clearly attributed to the (1, 1) SPP mode on the Ag/glass interface, which is strongly supported by the theoretical predictions based on Eq. 1. The first peak (*P1*), however, cannot be reproduced using the dispersion relations for SPP and RA modes. Chen *et al.*^34^ suggest that the slits making up the cross-shaped apertures can be treated as a bound charge oscillator state when illuminated with linearly polarized light having a shape resonance (or cut-off frequency)^51^ of:


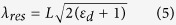


where *L* is the arm length. Stalzer *et al.*^52^ treated the cross-shaped aperture as a waveguide and calculated the TE and TM modes for different arm length-to-width ratios using the two-dimensional scalar Helmholtz equation. Based on this model, the cut-off frequency for the TE_10_ mode (which is the one that is situated in the spectral range studied here) is expressed as:


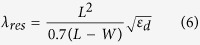


where *W* is the arm width. Using both the approaches of *Chen et al.* and *Stalzer et al.*, the cut-off frequency for the apertures used in this study is expected to occur in the range of 411 and 463 nm respectively. Thus, this LSP mode alone cannot explain the origin of the second peak situated in the red region of the spectrum.

From the experimental data in Fig. 2b, which summarizes the spectral evolution as the periodicity changes, another interesting aspect can be noted. The *P1* peak situated at the longer wavelengths has a distinct Fano-type asymmetric line-shape compared to the *P2* peak situated at shorter wavelengths. This asymmetry was described in terms of an interference between a continuum and a discrete state in a quantum mechanical study of the autoionization of atoms^53^. As the periodicity increases, the position of both peaks shifts to longer wavelengths, and the shape of the *P1* peak slowly changes into a Fano-type asymmetric line-shape as well. This might be explained by the fact that the LSPs couple with the electromagnetic field of the SPPs and RAs, leading to an asymmetric Fano-type line-shape of the spectrum. These LSP modes have a narrow energy dispersion situated in the short wavelength region, given by the relation^54^,^55^:


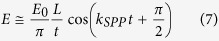


where *k*_*SPP*_ = 2π/*λ*_*SPP*_, *E*_*0*_ is the electrical field of the incident plane wave and *t* is the metal film thickness.

## Discussion

**Figure 7:**
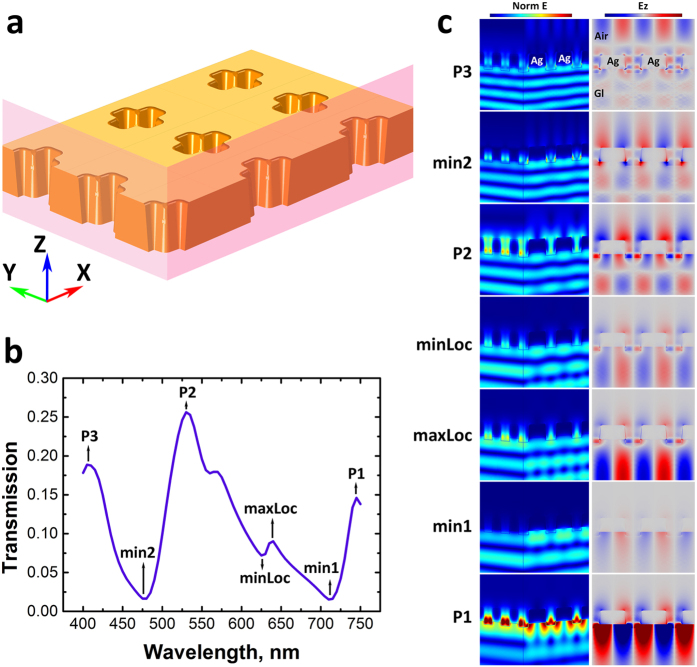
(**a**) Schematic of the aperture structure showing the cross-sectional planes (YZ and ZX) along which the normalized electrical field distribution (Norm E) are displayed in (**c**). (**b**) Calculated transmission through the structure corresponding to the sample S8 denoting the features (max and min) at which the electrical field distribution were displayed in (**c**). (**c**) Normalized electrical field distribution (Norm E) along the YZ and ZX planes, and amplitude of the field component normal to the metal surface (Ez) in ZX plane. All data are displayed for TM polarisation and normalized to the highest intensity range.

Using FEM simulation data, the electric field distributions were calculated and displayed for the maxima and minima at the cross-section of the aperture for sample S8 (Fig. 7).

The position of the features in Fig. 7b,c were assumed to correspond to the electric field distribution near the SPP, RA and LSP modes which are experimentally observed in the transmitted spectra and theoretically calculated (Fig. 6) in far-field. From Fig. 7c, it is clear that the electrical fields of the maxima associated with peaks *P1*, *P2* and *P3* have a different mechanism of far-field propagation. Peak *P3* at ~407 nm shows a strong localized mode within the aperture (noticeably visible in the amplitude of the field component normal to the metal surface, *Ez* plot), which might be attributed to the LSP. Indeed, the signature of this localized mode is expected to occur at ~411 nm and since all main SPP and RA modes occur at longer wavelengths, they do not interfere destructively with this LSP mode leading to a maxima in the spectra. The absence of any SPP and RA modes is also evident in the ‘Norm E’ intensity maps for the *P3* peak (Fig. 7c), where no localized electrical fields are observed at the Ag/glass or Ag/air interfaces. Based on the experimental results and the theoretical predictions, the feature (*min2*) at ~475 nm corresponds to the SPP(1, 0) Ag/air mode, which is observable as a strong electric field at the Ag/air interface. As this mode is close to the LSP resonant frequency, it is apparent that it interferes destructively with the LSP mode producing a minima in the spectra. At 525 nm (*P2* peak), the LSP and RA(1, 1) modes are no longer dominant modes, which freely allow far-field propagation of the SPP(1, 1) Ag/glass mode. The RA(1, 0) mode at 625 nm appears as a localized minimum (*minLoc*) in the spectra demonstrating that the SPP(1, 1) Ag/glass and RA(1, 0) modes destructively interfere. The SPP(1, 0) Ag/glass mode at ~707 nm (*min1*) shows up as a minimum in the spectra. Electrical fields (Norm E and Ez) in Fig. 7c reveal localized distributions at the Ag/glass interface with no far-field propagation through the apertures, which is deducted from the weak values of the electric field in far-field. Interestingly, at 745 nm (*P1* peak) a strong electric field distribution is observed at the aperture rims with an enhanced propagation, which could be attributed to a leaky SPP(1, 0) Ag/glass mode.

In conclusion, we have experimentally demonstrated the use of cross-shaped nano-apertures for continuous polarisation controlled color tuning in the visible range. This behaviour was achieved by fine-tuning the array periodicity, whilst keeping the geometry of individual apertures constant. The effect was demonstrated for both transverse electric and magnetic fields. Polarisation-induced sensitivity of the transmission spectra was also demonstrated for a nanometer-size, sub-wavelength set of aperture arrays. An anomalous set of maxima and minima in the transmission spectra were experimentally detected and compared to theoretical calculations which were used to interpret the underlying mechanisms of light-aperture interaction and far-field propagation. The role of LSPs is clearly dominant at shorter wavelengths, close to the cut-off frequency of the LSP mode, whilst the effect of SPPs and RA anomalies is apparent at longer wavelengths. The interferences of these modes led to a set of maxima and minima in the transmission spectra. Numerical simulations using FEM confirmed the experimental observations and fundamental theoretical predictions describing the propagation of LSP, SPP and RA modes and their interactions. Our results, which illustrate the potential of plasmonics for achieving polarisation-dependent, fine color control in the visible range, opens up new opportunities for dynamic nano-pixel color displays and high sensitivity bio-sensing.

## Methods

### Sample preparation and fabrication

For our study plasmonic devices based on the design in Fig. 1 were fabricated in a 150 nm thick Ag film with a 3 nm Ge adhesion layer. Both layers were deposited by electron-beam evaporation (Nanochrome II, Intlvac) at a 3 Å/s deposition rate on a semiconductor grade quartz substrate. Focused Ion Beam (FIB) lithography (Helios NanoLab 600 Dual Beam FIB-SEM, FEI) was employed to mill the apertures using 9.7 pA currents. Optical images were collected using an inverted optical microscope (Nikon Ti-U) at x100 magnification utilising a broad-band Xenon light source. Transmission spectra were acquired using the same set-up, employing a spectral analyser (IsoPlane SCT 320, Princeton Instruments) at 1200 gratings/mm. All spectra were normalized with respect to the bare quartz substrate using the patterned area as the region of interest.

### FEM analysis

FEM simulations were performed in COMSOL Multiphysics 5.1 using the Radio Frequency module^46^. The optical properties of Ag were taken from ref 48 and the dielectric permittivity of the quartz substrate was taken as 2.31. The modelled region consisted of a unit cell stack (quartz substrate, silver film with aperture and air) with a height of 2 μm. Floquet periodic boundary conditions were applied in the X and Y directions of the cell to simulate an infinite periodic array. The arms of the apertures were designed to be parallel to the periodicity direction. Plane wave illumination was used and the polarisation was adjusted by using the corresponding angle (0, 45° and 90 degrees for TM, 45° and TE polarisations respectively). For each polarisation, a parametric sweep was applied in the 400–750 nm spectral range with 5 nm resolution. The transmission spectra were calculated by integrating the Poynting vector over the exit surface and normalizing to that obtained in the absence of the Ag film. Figure 1a illustrates the conceptual design of the cross-shaped aperture arrays used in this study.

## Additional Information

**How to cite this article**: Balaur, E. *et al.* Continuously Tunable, Polarization Controlled, Colour Palette Produced from Nanoscale Plasmonic Pixels. *Sci. Rep.* **6**, 28062; doi: 10.1038/srep28062 (2016).
